# Investigation of growth characteristics and semimetal–semiconductor transition of polycrystalline bis­muth thin films

**DOI:** 10.1107/S2052252519015458

**Published:** 2020-01-01

**Authors:** Nan Wang, Yu-Xiang Dai, Tian-Lin Wang, Hua-Zhe Yang, Yang Qi

**Affiliations:** a Department of Materials Physics and Chemistry, School of Materials Science and Engineering, State Key Laboratory of Rolling and Automation, Northeastern University, Shenyang 110819, People’s Republic of China; b Department of Biophysics, School of Fundamental Sciences, China Medical University, Shenyang, Liaoning 110122, People’s Republic of China

**Keywords:** surface roughness, polycrystalline Bi thin films, low-melting metals, molecular beam epitaxy, semimetal–semiconductor transitions, structure-zone models

## Abstract

The preferred orientation growth characteristics and surface roughness of polycrystalline bis­muth (Bi) thin films fabricated on glass substrates using the molecular beam epitaxy method were investigated at temperatures ranging from 18 to 150°C. The crystallization and morphology were analyzed in detail and the polycrystalline metal film structure-zone model (SZM) was modified to fit the polycrystalline Bi thin film. The boundary temperature between Zone T and Zone II in the SZM shifted to higher temperatures with the increase in film thickness or the decrease of growth rate. Furthermore, the effect of the thickness and surface roughness on the transport properties was investigated, especially for Bi thin films in Zone II. A two-transport channels model was adopted to reveal the influence of the film thickness on the competition between the metallic surface states and the semiconducting bulk states, which is consistent with the results of Bi single-crystal films. Therefore, the polycrystalline Bi thin films are expected to replace the single-crystal films in the application of spintronic devices.

## Abstract 2

The effect of substrate temperature, growth rate and film thickness on the crystallographic characteristics of polycrystalline Bi thin films by molecular beam epitaxy and semimetal–semiconductor transition are discussed. Meanwhile, a structure-zone model and a two-transport-channels model are introduced to explain the crystal growth characteristics and the semimetal–semiconductor transition.

## 1. Introduction   

Semimetal Bi is one of the most well documented materials in solid-state physics because of its unique physical properties, such as low carrier density (∼3 × 10^17^ cm^−3^), small effective masses (*m*_e_ ≃ 10^−3^), long mean-free path (30 nm), highly anisotropic fermi surface and small band overlap at cryogenic temperature (∼38 meV) (Marcano *et al.*, 2010 ▸; Hofmann, 2006 ▸; Liao *et al.*, 2014 ▸; Xiao *et al.*, 2012 ▸). The quantum effect of Bi thin films is remarkable because of their semimetal–semiconductor (SMSC) transition (Xiao *et al.*, 2012 ▸; Hoffman *et al.*, 1993 ▸; Chu, 1995 ▸; Lu *et al.*, 1996 ▸). In 1967, Sandomirskii first predicted that 30 nm was the critical thickness for the SMSC transition of Bi thin films caused by quantum-size effect (QSE) (Sandomirskii, 1967 ▸). Ast & Höchst observed that the electronic states of bulk Bi(111) (rhombohedral crystallographic system) were composed of the surface states using angle-resolved photoelectron spectroscopy (Ast & Höchst, 2001 ▸). Nagao *et al.* found that the property of metal surface states was identical with varied thicknesses and then confirmed that the metal states were formed by spin-orbital-split surface states (Nagao *et al.*, 2004 ▸; Hirahara *et al.*, 2006 ▸). In addition, Bi is also a key element of many functional materials such as topological insulators, thermoelectric materials, valleytronic materials and superconductors (Qi & Zhang, 2011 ▸; Poudel *et al.*, 2008 ▸; Zhu *et al.*, 2011 ▸, 2017 ▸; Zhang & Qi, 2011 ▸). Therefore, Bi has recently attracted renewed interest and has become one of the hot research topics.

The growth modes of high-quality thin films are usually divided into epitaxial growth and preferred orientation of growth. The key factor of epitaxial growth is the lattice matching relationship between the films and the substrates (Kokubo *et al.*, 2015 ▸; Cho *et al.*, 1998 ▸; Plaza *et al.*, 2012 ▸). In contrast, the preferred orientation growth is mainly affected by the surface energy rather than the lattice matching. Xiao *et al.* prepared epitaxial Bi(111) thin films on BaF_2_(111) substrates using the molecular beam epitaxy (MBE) method and explained the SMSC transition according to the competitions of metallic surface states and semiconducting bulk states (Xiao *et al.*, 2012 ▸). The two-channel coupling transport between the surface states and the bulk states was confirmed by *in situ* magnetic measurements on ultrathin single-crystal Bi(111) thin films epitaxially grown on an Si(111) (7 × 7) surface (Aitani *et al.*, 2014 ▸).

In recent years, research on the preferred orientation of Bi thin films has made some progress (Marcano *et al.*, 2010 ▸, 2013 ▸; Liao *et al.*, 2014 ▸; Fedotov *et al.*, 2015 ▸, 2016 ▸). Wu found that Bi thin films with (111) preferred orientation could be obtained on glass substrates at temperatures ranging from −40 to 93°C *via* pulse laser deposition, while the orientation was random when the substrate temperature rose to 200°C (Wu & Chern, 2008 ▸). Nakada found that the orientations of Bi thin films prepared using ionized clusters changed with the rising substrate temperatures (from 30 to 105°C) (Nakada *et al.*, 1996 ▸). Namba observed that the surface roughness on glass substrates prepared by a thermal evaporation method was directly affected by the high deposition rate and the substrate temperature (Namba & Mori, 1975 ▸).

As mentioned above, many studies have focused on the effects of substrate types and temperatures on the microstructure and transport properties of epitaxial Bi thin films. However, the mechanism of the preferred orientation growth, the effect of growth rate on the film microstructure and the SMSC transition of preferred orientation polycrystalline Bi thin films, to the best of our knowledge, have rarely been reported.

In this article, polycrystalline Bi thin films with preferred orientation were fabricated on glass substrates *via* the MBE method. A structure-zone model (SZM) for low-melting-point metal polycrystalline Bi films is proposed for the first time. The effects of substrate temperature on the preferred orientation, microstructure and surface morphology, as well as the influence of film-surface roughness and thickness on the transport properties, have been investigated in detail.

## 2. Experimental   

Bi thin films were fabricated on glass substrates using the MBE method. The purity of the raw material was higher than 99.99%. The base pressure of the growth chamber was below 2.0 × 10^−7^ Pa. The thin films were grown at various substrate temperatures (18, 30, 40, 50, 60, 70, 80, 90, 100, 110, 120, 130, 140 and 150°C) using different deposition rates (1.61, 0.47, 0.19 and 0.14 Å min^−1^). The crystal properties were determined by X-ray diffraction (XRD, SmartLab) with a Cu *K*α_1_ X-ray radiation source (λ = 1.540562 Å). The angular scans were realized in a Bragg–Brentano focusing configuration (θ/2θ) with 2θ ranging from 10 to 80° and a scanning rate of 4° min^−1^. The morphology of films was observed using a field-emission scanning electron microscope (FESEM, Zeiss, ULTRAPLUS) and an atomic force microscope (AFM, Nanosurf EasyScan2). The in-plane orientations and the grain sizes of the resulting thin films were characterized by electron backscatter diffraction (EBSD, JSM-7800F Oxford EBSD NordlysNano) on an FESEM equipped for this. The transport properties were characterized using a physical property measurement system (PPMS, Quantum Design, DynaCool-9) equipped with a high-precision rotator and a magnetic field up to 9 T.

## 3. Results and discussion   

**Figure 1:**
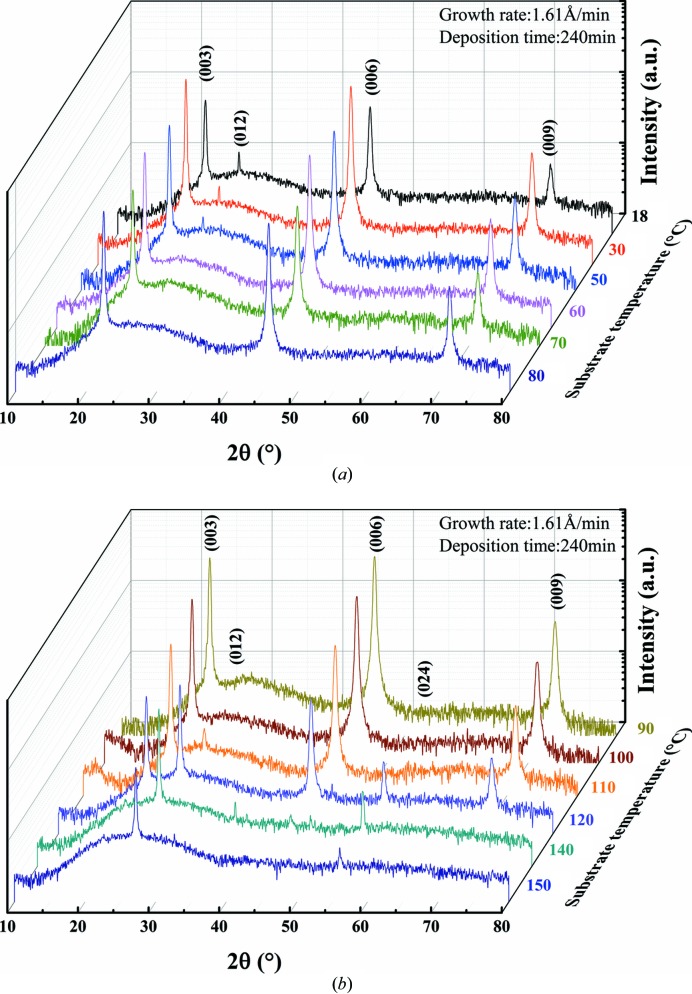
XRD patterns of Bi thin films on glass at different substrate temperatures (the growth rate is 1.61 Å min^−1^ and the deposition time is 240 min). (*a*) The XRD patterns of Bi thin films at substrate temperatures of 18, 30, 50, 60, 70 and 80°C and (*b*) the XRD patterns of Bi thin films at substrate temperatures of 90, 100, 110, 120, 140 and 150°C.

The substrate temperature is one of the important factors that determine the crystal growth characteristics of thin films. Fig. 1 ▸ shows the XRD patterns of Bi thin films prepared at substrate temperatures ranging from 18 to 150°C with a constant growth rate of 1.61 Å min^−1^ and a deposition time of 240 min. Bi crystals have been indexed in many ways (Hirahara *et al.*, 2006 ▸). The [0001] trigonal axis in hexagonal notation corresponding to the [111] axis in rhombohedral notation is applied in this article because of its mathematical accessibility and convenience for diffraction experiments. The XRD patterns mainly exhibit the (003), (006) and (009) diffraction peaks during the substrate temperatures ranging between 18 and 100°C according to JCPDS card No. 05-0519 [space group 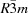 (No. 166), *a* = *b* = 4.546 Å and *c* = 11.86 Å]. The (012) diffraction peak gradually decreases as the substrate temperature rises from 18 to 50°C but there is no (012) diffraction peak for the temperature range from 60 to 100°C. The (012) diffraction peak gradually increases as the substrate temperature rises above 110°C and (00*l*) diffraction peaks start to decrease at 120°C. As the substrate temperature rises up to 150°C, only (012) and (024) diffraction peaks exist in the XRD pattern, while the (00*l*) diffraction peaks disappear. Therefore, the preferred orientation growth of the Bi thin films changes from 18 to 150°C.

**Figure 2:**
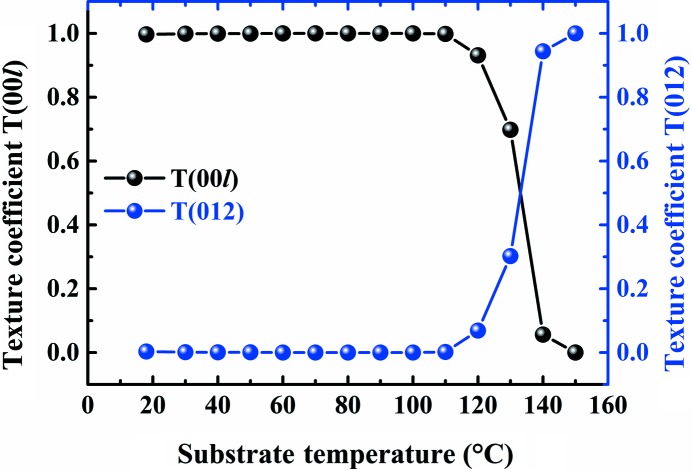
Texture coefficient variation of *T*(00*l*) and *T*(012) at substrate temperatures estimated from θ–2θ scans (the growth rate is 1.61 Å min^−1^ and the deposition time is 240 min).

The texture coefficient can be analyzed based on the Harris method (Harris, 1952 ▸) as follows, 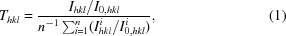
where *T_hkl_* is the texture coefficient of the (*hkl*) crystal plane, *I_hkl_* is the corresponding diffraction intensity, *n* is the crystal plane number and *I*_0,*hkl*_ is the corresponding diffraction peak intensity of the standard JCPDS database. *T_hkl_* represents the variety of the grain preferred orientation along the (*hkl*) plane. A larger *T_hkl_* value means a stronger preferred orientation along the normal of the (*hkl*) plane and *vice versa*. The texture coefficients of (003) and (012) are used to represent the two kinds of preferred orientations because (003), (006) and (009) planes belong to the (003) family while (012) and (024) planes belong to the (012) family. The *T_hkl_* of different substrate temperatures are shown in Fig. 2 ▸. When the substrate temperature is in the range from 18 to 100°C, the (00*l*) texture coefficient approaches 1 while the (012) texture coefficient approaches 0. As the substrate temperature rises from 110 to 150°C, the (00*l*) texture coefficient decreases sharply to 0 and the (012) texture coefficient correspondingly increases from 0 to 1. These results indicate that the preferred orientation of the Bi thin films fabricated using the MBE method changes from (003) to (012) with substrate temperatures rising from 18 to 150°C. The ordering transfers from (003) to (012) with a hexagonal lattice texture (Grantscharova, 1993 ▸). The transformation of preferred orientation could intrinsically be attributed to the surface energy of Bi. The Bi(003) plane has three covalent bonds while the Bi(012) plane has only two covalent bonds and leaves a dangling bond on the surface (Koroteev *et al.*, 2008 ▸). Additionally, the Bi(003) plane has a larger atom density and lower entropy than those of the Bi(012) plane. Thus, Bi thin films present (003) preferred orientation at relatively low temperature. With the increase in temperature, the (00*l*) plane gradually becomes unstable (Nagao *et al.*, 2004 ▸), so the preferred orientation of Bi thin films changes from the (00*l*) to the (012) planes.

**Figure 3:**
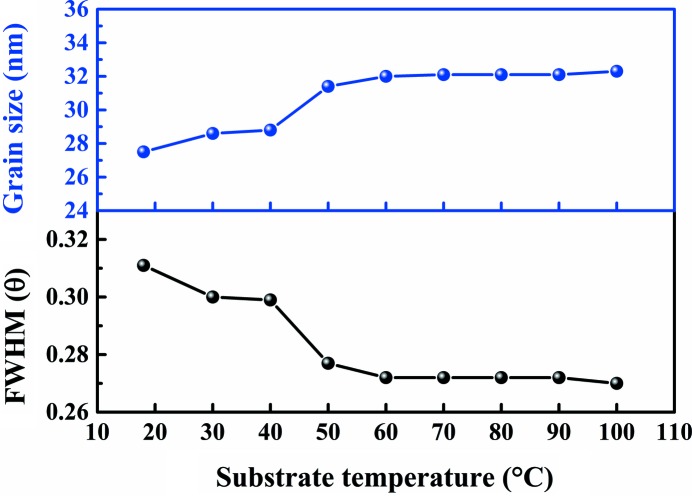
The FWHM and out-of-plane grain size as a function of Bi thin films for substrate temperatures (the growth rate is 1.61 Å min^−1^ and the deposition time is 240 min).

Fig. 3 ▸ shows the temperature dependence of the full width at half-maximum (FWHM) and out-of-plane grain size of the (003) diffraction peak. As observed in the figure, the FWHM initially decreases with the increasing temperature until ∼60°C where the FWHM becomes constant. The out-of-plane grain size increases then also becomes essentially constant above 60°C. In the substrate temperature range from 18 to 60°C, multiple-grain out-of-plane Bi thin films exist. The columnar crystal growth normal to the surface results in only one grain thick over the 60–100°C substrate temperature range. The normal grain size obtained by XRD is slightly smaller than the film thickness because the grain-size value obtained by XRD is a statistical value.

**Figure 4:**
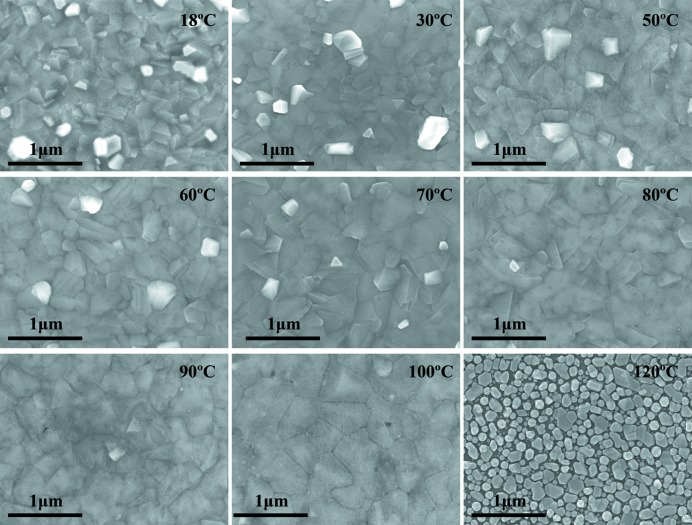
SEM images of Bi thin films at various substrate temperatures, *i.e.* 18, 30, 50, 60, 70, 80, 90, 100 and 120°C (the growth rate is 1.61 Å min^−1^ and the deposition time is 240 min).

**Figure 5:**
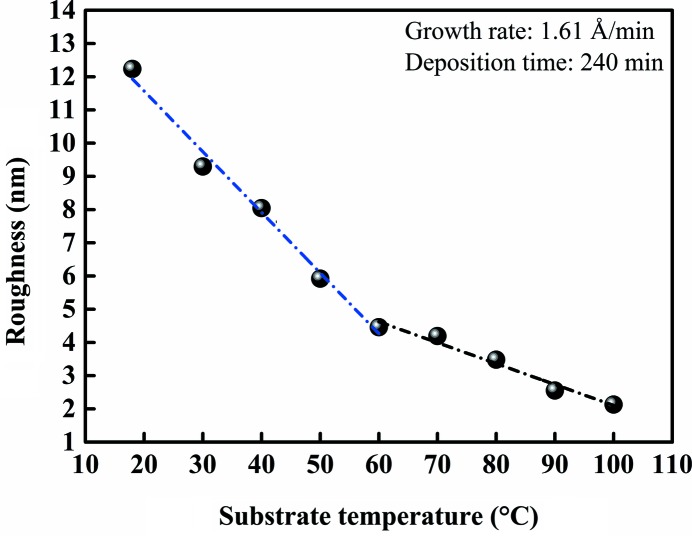
The RMS roughness as a function of Bi thin films for substrate temperatures (the growth rate is 1.61 Å min^−1^ and the deposition time is 240 min).

Growth characteristics of polycrystalline thin films are affected by the substrate temperature and the metal melting point. Fig. 4 ▸ shows the SEM surface topographies of Bi thin films prepared at different substrate temperatures ranging between 18 and 120°C. As the substrate temperature increases, the larger raised grains on the surface of the film become less and smoother. The grain boundaries are clear at the substrate temperatures below 70°C and become smooth above 70°C. Because of the change in preferred orientation from (003) to (012) above 110°C, the surface morphology significantly changed from layered growth to island growth as shown in Fig. 4 ▸. Fig. 5 ▸ shows the dependence of corresponding Bi thin films surface roughness on the substrate temperature (area size range: 2 × 2 µm). As shown in Fig. 5 ▸, the surface roughness of the Bi thin films sharply decreased at the substrate temperatures from 18 to 60°C. The surface roughness also slowly decreased at the substrate temperatures from 60 to 100°C. Therefore, the surface roughness gradually decreases with the rising of substrate temperature. It is clear that with the increase in substrate temperature the in-plane diffusion of atoms leads to sufficient grain growth and smooth grain boundaries so that the film becomes smooth.

**Figure 6:**
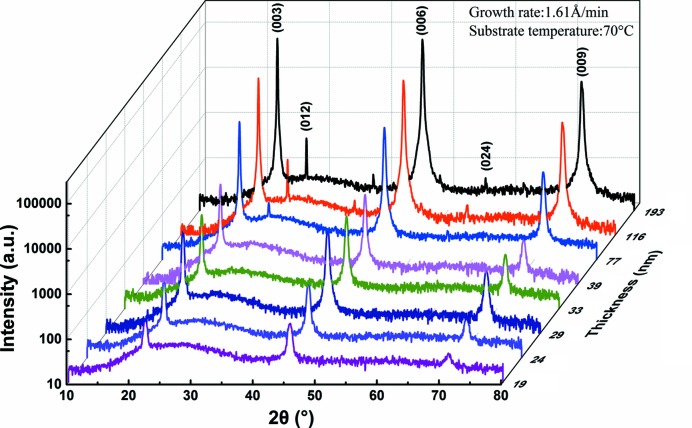
XRD patterns of Bi thin films with various thicknesses. The thicknesses are 19, 24, 29, 33, 39, 77, 116 and 193 nm (the growth rate is 1.61 Å min^−1^, the substrate temperature is 70°C and *T*_s_/*T*_m_ = 0.63).

In order to further optimize the quality of the Bi film, the effects of film thickness and growth rate need to be discussed in detail at a specific temperature. The specific temperature was chosen to be 70°C since the Bi film had high texture characteristics and clear surface morphology at this temperature. Fig. 6 ▸ shows the XRD patterns of Bi thin films with different thicknesses derived at the same growth rate (1.61 Å min^−1^) (the film thicknesses were 193, 116, 77, 39, 33, 29, 24 and 19 nm). There is no change in the preferred orientation with thicknesses up to 77 nm. At a thickness of 77 nm and greater, the (012) crystal plane diffraction peak starts to appear. The appearance of this peak corresponds to a reduction of the texture coefficient for the (003) orientation.

**Figure 7:**
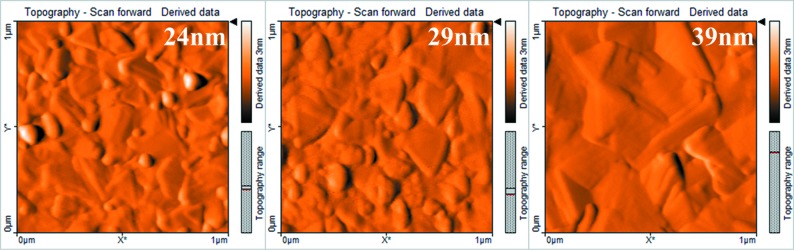
AFM images (1 × 1 µm) of Bi thin films with different thicknesses of 24, 29 and 39 nm.

Fig. 7 ▸ shows the AFM surface topography images of Bi thin films with different thicknesses. The triangular shapes of the Bi thin film surface become more obvious with the increase in film thickness. Namely, the grain does not start to diffuse completely and maintains its primary crystal structure at 70°C. The average grain size of in-plane crystals in EBSD statistic increases with the increase in thickness of films. A few grains with (012) orientation appear in thicker films because of the relatively faster growth rate. The results of XRD and EBSD analysis indicate that there is a significant effect of film thickness on the in-plane/out-of-plane grain size. Compared with the decrease of growth rate, the increase in film thickness has a much greater effect on the surface roughness of the film and is independent of the substrate type (Koseva *et al.*, 2012 ▸).

**Figure 8:**
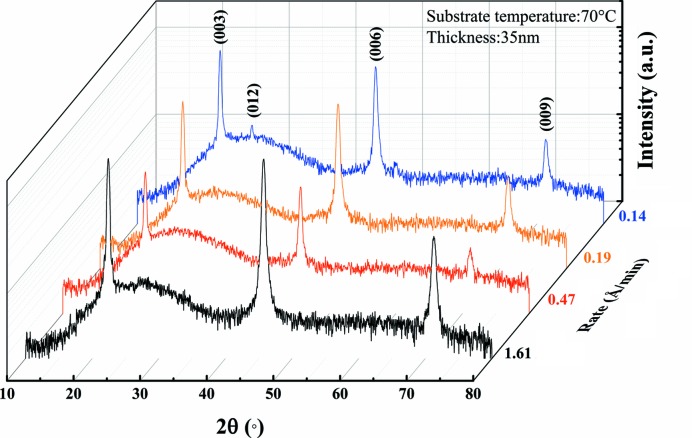
XRD patterns of Bi thin films with different growth rates at 0.14, 0.19, 0.47 and 1.61 Å min^−1^ (the thickness is 30 nm, the substrate temperature is 70°C and *T*_s_/*T*_m_ = 0.63)

Fig. 8 ▸ shows the XRD patterns of Bi thin films (with thicknesses of around 30 nm and a substrate temperature of 70°C) deposited at growth rates of 1.61, 0.47, 0.19 and 0.14 Å min^−1^. Bi(00*l*) diffraction peaks are observed in the XRD patterns, which means that the preferred orientation of the films is independent of the different growth rates. However, when the growth rate is reduced to 0.14 Å min^−1^, a weak (012) diffraction peak appears at 70°C.

**Figure 9:**
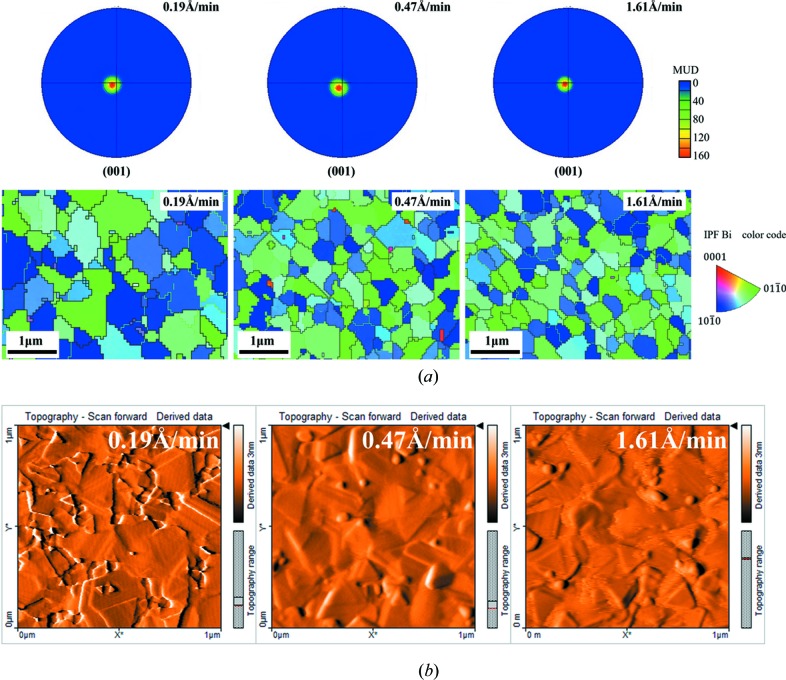
The microstructure of the low-melting-point metal polycrystalline Bi thin films with different growth rates (the thickness is 30 nm and the substrate temperature is 70°C). (*a*) EBSD images of Bi thin films (thickness 30 nm) with different growth rates at 0.19, 0.47 and 1.61 Å min^−1^. The color code shows the crystallographic orientation with respect to [0001]. (*b*) AFM images (1 × 1 µm) of Bi thin films with different growth rates at 0.19, 0.47 and 1.61 Å min^−1^.

Fig. 9 ▸(*a*) shows the EBSD pole figures and inverse pole figure (IPF) orientation patterns of Bi thin films grown at 0.19, 0.47 and 1.61 Å min^−1^. The high concentration of red near the center in the pole figures indicates that the grains have a highly preferred (00*l*) out-of-plane orientation. The random green and blue grains in the IPFs show these grains have a random in-plane orientation. The orientation difference is defined as the large-angle grain boundary when the difference between the two grains is greater than 15°. The average in-plane grain size in the EBSD statistic increases as the growth rate decreases, which is in accordance with the result of XRD. This is because the low growth rate has sufficient in-plane diffusion time resulting in lateral grain growth. The grain-boundary density of the films with low growth rate is less than the films with high growth rate.

In Fig. 9 ▸(*b*), AFM morphology patterns of these Bi thin films are analyzed. The morphology topography decreases as the growth rate increases. The surface roughness decreases with an increase in the growth rate, which is related to the grain size. This phenomenon can be attributed to the Ostwald ripening mechanism (Ostwald, 1897 ▸). The Bi grains have sufficient time for diffusion after nucleation at a low growth rate, and large nuclei combine with small nuclei to reduce the interfacial energy of the system, which leads to the increase in grain size. When the growth rate is high, the low diffusion energy and the incomplete grain growth lead to the small grain size. Meanwhile, the insufficient atom diffusion reduces the surface topography of the Bi thin films.

The schematic representation for the SZM was proposed by Movchan & Demchishin (1969 ▸). A metal film deposited on a substrate with a temperature gradient has three characteristic structure zones with the boundary temperatures *T*_s_/*T*_m_ = 0.3 and *T*_s_/*T*_m_ = 0.5, where *T*_s_ is the substrate temperature and *T*_m_ is the metal melting point (in kelvin) (Movchan & Demchishin, 1969 ▸; Higo *et al.*, 2006 ▸). Then, in the aluminium polycrystalline film, Zone T is proposed between Zone I and Zone II, and the temperature interval 0.2 < *T*_s_/*T*_m_ < 0.4 (Barna & Adamik, 1998 ▸). In Zone I, the surface diffusion of adatoms is too slow to diffuse into the shadowed regions. In Zone T, the structure is inhomogeneous along the film thickness. The films in Zone II have the smoothest surfaces; therefore, the surface diffusion of adatoms is sufficient, which leads to surface recrystallization in this zone. The films in Zone III have a polyhedral surface structure which consists of equated grains with bright surfaces because of the dominant influence of bulk diffusion and recrystallization on the film structure (Barna & Adamik, 1998 ▸; Thornton, 1977 ▸; Thompson, 2000 ▸).

Though the SZM explains the general evolution in the surface morphology of the metal films, low-melting-point metal films have individual surface morphology and roughness characteristics. The low-melting metal polycrystalline Bi films prepared at *T*_s_/*T*_m_ = 0.55–0.70 showed the individual characteristic surface morphology similar to that of Zone II mentioned above (Higo *et al.*, 2006 ▸), but no further analysis was researched. Thus, boundary temperature was redefined in the Bi thin films as follows. Firstly, the SZM is still dependent on *T*_s_/*T*_m_, where *T*_s_ is the substrate temperature and *T*_m_ is the melting point of the film material (*T*_m_ = 544.3 K for Bi). The *T*_s_/*T*_m_ value was determined by the self-surface diffusion level and it affected the mobile degree of grain boundaries, which led to the lateral grain growth on the substrate. For the low-melting polycrystalline Bi films, an improved structural zone is proposed, where Zone T (*T*_s_/*T*_m_ < 0.61) is the competitive texture with low adatom mobility, Zone II (0.61 < *T*_s_/*T*_m_ < 0.68) is the stable texture with surface diffusion and Zone III (*T*_s_/*T*_m_ > 0.68) is the restructuration texture with bulk diffusion.

Zone T is characterized by small grain size and complete out-of-plane texture in the films. The grain size becomes large because of surface diffusion of adatoms, and the film surfaces have individual characteristic morphology and roughnesses as *T*_*s*_ increases. Zone II is characterized by homogeneous grain size and constant out-of-plane texture of the film. As the substrate temperature increases, the self-surface diffusion increases remarkably, which enables the film to grow smoothly and surface roughness to decrease. The limited grain-boundary migration gradually transforms to the movable grain boundaries, resulting in lateral grain growth on the substrate. Through detailed analysis of the texture and surface topography above, the boundary temperature of the polycrystalline Bi thin films between Zone T to Zone II is not a fixed point. This indicates that the increasing thickness of the film and the growth-rate reduction causes Zone II to shift to Zone T. Zone III is characterized by the polyhedron crystal morphology and the reconstruction texture of the film, and finally the preferred orientation is controlled by the competition of the interface and surface energy.

**Figure 10:**
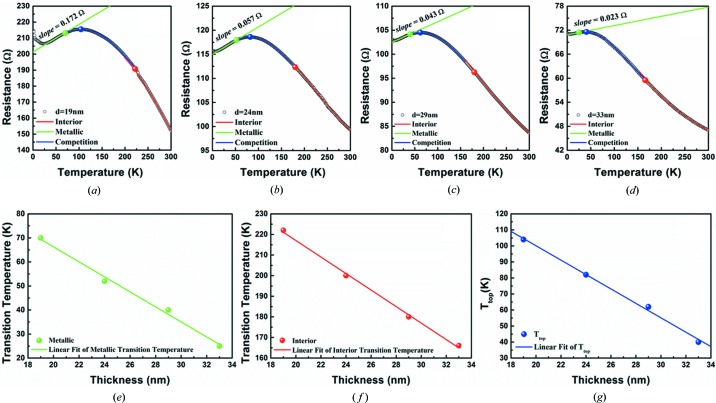
The temperature dependence for resistance of Bi thin films at thicknesses of (*a*) 19 nm, (*b*) 24 nm, (*c*) 29 nm and (*d*) 33 nm. The red line represents the e-index fitting, the green line represents the line fitting and the blue line represents two-transport channels fitting. Furthermore, (*e*)–(*g*) are the feature transition temperature points extracted from (*a*)–(*d*) and represent transition temperature as a function of sample thickness. (*e*) The deviation metal-surface-states point (green solid symbols), (*f*) the deviation semiconductor-bulk-states point (red solid symbols) and (*g*) the metal-surface-states transition temperature *T*_top_ corresponding to the maximum resistance in the metal and bulk states competition.

In Zone II, the polycrystalline Bi thin films with strong preferred orientation were prepared to study the basis of the SMSC transition and the stability of the device. The SMSC transition of Bi thin films was also affected by the quantum-well effect (Xiao *et al.*, 2012 ▸). Additionally, the film thickness limits the research on the surface states of Bi thin films because of the competition between the bulk states and the surface states. The resistance curves and their fittings of four thinner films in Zone II with noticeable SMSC transition are shown in Figs. 10 ▸(*a*)–10 ▸(*d*). At the high-temperature stage (red line), the resistance is governed mainly by the semiconductor channel and increases exponentially as a function of temperature. While at the low-temperature stage (green line), the contribution from the semiconductor part can almost be neglected and the resistance is governed mainly by the metal channel, which increases linearly as a function of temperature. As a result, the resistance is governed by the parts of both the semiconductor and metal at the stage of competition transition temperature (blue line). Therefore, a two-transport channels model should be used to describe the resistance of competition states as follows. 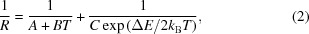
where *k*_B_ denotes the Boltzmann factor, and *A*, *B* and *C* are the parameters. 1/(*A* + *B* *T*) describes the metallic transport including electron–phonon scattering and 1/[*C* exp(Δ*E*/2*k*_B_ *T*)] describes the semiconductor transport that accounts for thermally excited carriers.

A maximum resistance value can be found in the competition of metal and bulk states, which is defined as the metal-surface-states transition temperature *T*_top_. These three different series of data have a linear relationship with the film thickness. The semiconductor-state temperature (red symbol), the metal-state temperature (green symbol) and the *T*_top_ temperature (blue symbol) as a function of the Bi thin film thickness are analyzed in Figs. 10 ▸(*e*)–10 ▸(*g*). The corresponding transition temperature decreases as the film thickness increases. The thickness directly affects the SMSC transition because the Bi thin films have shorter mean-free paths than that of Bi bulk because of the stronger phonon scattering or impurity inside the film (Xiao *et al.*, 2012 ▸; Hirahara *et al.*, 2007 ▸). Therefore, the competition between surface states and the bulk states can explain the SMSC transition in polycrystalline Bi thin films, which is highly consistent with the results of Bi single-crystal films (Xiao *et al.*, 2012 ▸; Barna & Adamik, 1998 ▸).

**Figure 11:**
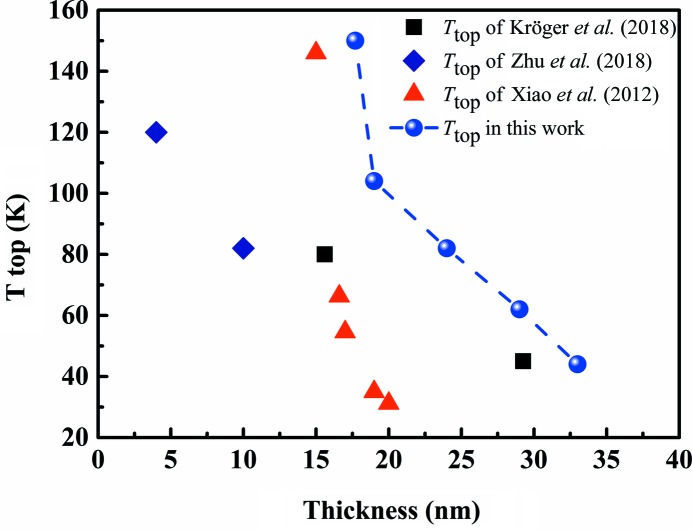
Comparison of *T*_top_ in single-crystal and polycrystalline Bi thin films. The variation of *T*_top_ of Bi thin films with different thicknesses. The squares represent the *T*_top_ of Bi(111) film grown on Si(111) in the work by Kröger *et al.* (2018 ▸), the rhombuses represent the *T*_top_ of Bi(111) film grown on Si(111) in the work by Zhu *et al.* (2018 ▸), the triangles represent Bi(111) film grown on BaF_2_(111) in the work by Xiao *et al.* (2012 ▸) and the dots represent Bi(00*l*) film grown on glass in this work.

The *T*_top_ of single-crystal and polycrystalline Bi thin films is shown in Fig. 11 ▸. In Fig. 11 ▸, the *T*_top_ of Bi(111) single-crystal thin film epitaxially growing on Si(111) substrate is represented as black squares in the work by Kröger *et al.* (2018 ▸), the *T*_top_ of Bi(111) single-crystal thin film epitaxially growing on Si(111) substrate is represented as blue rhombuses in the work by Zhu *et al.* (2018 ▸) and the *T*_top_ of Bi(111) single-crystal thin film epitaxially growing on BaF_2_(111) substrate is represented as red triangles in the work by Xiao *et al.* (2012 ▸). In this work, the *T*_top_ temperature is represented (blue dots) as a function of the thickness of Bi(00*l*) polycrystalline films on glass substrate. The deviation of the 19 nm film is attributed to the surface states. The surface states make a dominant contribution and are independent of the film thickness. The temperature then has a great influence on the surface states (Aitani *et al.*, 2014 ▸). The polycrystalline thin film was found to have the consistent SMSC transition as the single crystal. The grain boundary had no negative effect on it.

**Figure 12:**
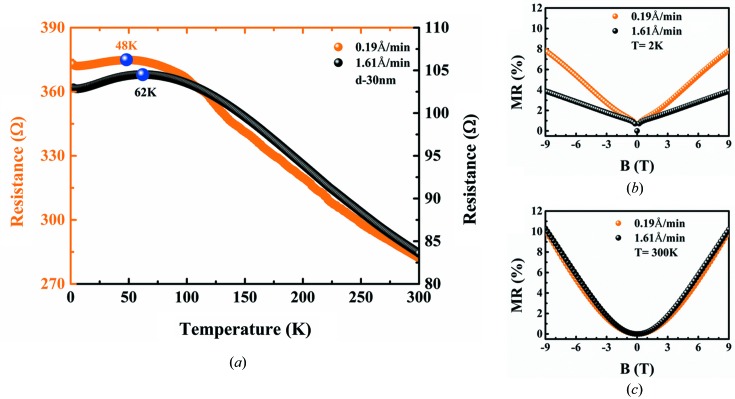
(*a*) Growth-rate dependence for resistance of Bi thin films (30 nm) at 0.19 and 1.61 Å min^−1^. Blue spheres indicate the transition temperature points *T*_top_. (*b*) Normalized MR as a function of magnetic field *B* = −9 T – 9 T at *T* = 2 K, comparing growth rate at 0.19 and 1.61 Å min^−1^. Here MR = [*R*(*B*) − *R*(0)]/*R*(0) × 100, where *R*(*B*) and *R*(0) are the resistance with and without the magnetic field *B*, respectively. (*c*) Normalized MR as a function of magnetic field *B* = −9 T – 9 T at *T* = 300 K, comparing growth rate at 0.19 and 1.61 Å min^−1^.

Fig. 12 ▸(*a*) shows the effect of 30 nm Bi films with different growth rates on the electrical properties in Zone II. Compared with the film prepared at low growth rate, the film with fast growth rate has higher *T*_top_ because the in-plane grain size is smaller which results in the smoother surface. The grain size changes the competition ratio of the surface states and bulk states in a way that affects the film surface roughness and ultimately has an impact on the SMSC transition. Figs. 12 ▸(*b*) and 12 ▸(*c*) show the magnetic field dependence of the normalized magnetoresistance (MR) at 2 and 300 K. Here, MR = [*R*(*B*) − *R*(0)]/*R*(0) × 100, where *R*(*B*) and *R*(0) are the resistance with and without the magnetic field *B*, respectively. At *T* = 300 K, the MR ratio is about 10% at *B* = 9 T. While at *T* = 2 K, MR is observed to be almost linearly proportional to the magnetic field and the MR ratio of film with lower growth rate is about twice as fast as that at *B* = 9 T. It is well known that the magnetoresistance effect of the Bi thin film is related to the grain size because small grains cause a small mean-free path, thus further resulting in a small MR effect (Yang *et al.*, 1999 ▸). Hence, the surface roughness of the Bi thin film is the key factor that affects the SMSC transition rather than the density of the grain boundaries.

## 4. Conclusions   

In summary, high-quality polycrystalline Bi thin films on glass substrates were prepared using the MBE method. The Bi thin films with (00*l*) preferred orientation were obtained at substrate temperatures ranging from 18 to 100°C. The preferred orientation changed from (00*l*) to (012) when the substrate temperature exceeded 110°C, which was attributed to the different surface energy between (00*l*) and (012) planes. The kinetic results indicated that atomic diffusion did not change the preferred orientation. Therefore, the surface energy was the intrinsic inducing factor for the formation of polycrystalline Bi thin films with preferred orientation. The SZM of the low-melting polycrystalline Bi films was optimized. Furthermore, the low growth rate and the thickness increase led to a shift from Zone II to Zone T. The surface roughness and thickness of polycrystalline Bi thin films also affected the SMSC transition, which was induced by the competition between the semiconductor states and the metal surface states. The experimental results could be reasonably interpreted based on the two-transport-channel model, which were consistent with single crystals. Therefore, in the transportation process the in-plane grain boundary did not affect the transition of the semiconductor states and the metal surface states. The contribution of this work is to improve the study of the surface states of polycrystalline Bi thin films. Thus, the small surface roughness of Bi thin film is highly beneficial for protecting its metallic surface states and for its practical uses in Bi-film-based microelectronic and spintronic devices.
